# Zinc finger protein Y - linked as a potential biomarker for autoimmune hepatitis and multiple sclerosis which overlap with immune infiltration

**DOI:** 10.1038/s41598-026-43283-4

**Published:** 2026-03-27

**Authors:** Jian Liu, Di Guo, Meng Pu, Xin Li, Ying Xiao, Zi-wei Zhang, Yi-bin Tang, Yang Liu, Cun-gen Ma, Qing Wang

**Affiliations:** 1 Research Center of Neurobiology, The Key Research Laboratory of Benefiting Qi for Acting Blood Circulation Method to Treat Multiple Sclerosis of State Administration of Traditional Chinese Medicine, Shanxi University of Chinese Medicine, Jinzhong, 030619 Shanxi China; 2 Caidian District People’s Hospital, Wuhan, 430100 Hubei China; 3 College of Basic Medical Sciences, Shanxi University of Chinese Medicine, Jinzhong, 030619 PR China

**Keywords:** ZFY gene, Autoimmune hepatitis, Multiple sclerosis, Bioinformatics analysis, Immune infiltration analysis, PI3K/Akt pathway, Immunology, Autoimmunity

## Abstract

Autoimmune Hepatitis (AIH) and Multiple Sclerosis (MS) are chronic inflammatory diseases with abnormal immune responses. This study aims to identify common biomarkers for AIH and MS using bioinformatics analysis. Gene expression data of AIH (GSE159676) and MS (GSE131279 and GSE131281) were obtained from the GEO database. Differentially Expressed Genes (DEGs) were identified using the limma package in R. Kyoto Encyclopedia of Genes and Genomes (KEGG) analysis, Protein-Protein Interaction (PPI) network, and machine learning algorithm Least Absolute Shrinkage and Selection Operator (LASSO) regression were used to evaluate potential biomarkers. The common biomarker gene Zinc Finger Protein Y-linked (ZFY) was identified. KEGG analysis showed significant enrichment of the Phosphatidylinositol 3-Kinase/Protein Kinase B (PI3K/Akt) pathway in both diseases. LASSO regression identified ZFY as a potential diagnostic marker, with decreased expression in both AIH and MS groups. Single-gene immune infiltration analysis indicated a significant association between ZFY expression and immune cell infiltration levels. Experimental validation in ConA-induced hepatitis and CPZ-mediated demyelination model mice further verified the diagnostic potential of ZFY. This study reveals the potential of ZFY as a biomarker for AIH and MS, highlighting its role in the PI3K/Akt pathway and immune infiltration. These findings provide new insights into the common pathological mechanisms of AIH and MS and suggest potential targets for future therapeutic strategies.

## Introduction

Autoimmune diseases (AIDs) are a group of chronic diseases caused by abnormal immune responses^1^. Among them, autoimmune hepatitis (AIH) and multiple sclerosis (MS) are two typical representatives. AIH, on one hand, is a chronic liver inflammatory disease mediated by autoimmune responses^2^. It is characterized by hypergammaglobulinemia, positive autoantibodies, and responses to immunosuppressive therapies^3^. MS, on the other hand, is a chronic neuroinflammatory disease characterized by demyelination and axonal damage in the central nervous system (CNS), severely affecting the quality of life of patients^4^.

When exploring the pathogenesis of AIH and MS^5,6^, we found some remarkable common features, especially regarding the abnormal activation and dysregulation of the immune system. The core mechanism of both AIH and MS is the autoimmune response. In AIH, the immune system mistakenly recognizes liver tissues as foreign substances, triggering an inflammatory response that leads to hepatocyte damage and dysfunction. In MS, the immune system attacks CNS, particularly the medulla and white matter of the brain, causing conduction disorders and neurological deficits. Both diseases involve the abnormal activation of T cells and B cells, the over - release of cytokines, and the disruption of immune tolerance mechanisms^7^. Clinical and epidemiological observations—simultaneous or sequential onset of AIH and MS and a higher-than-expected comorbidity—suggest shared pathogenic roots^8,9^. These parallels are reinforced by overlapping genetic risk loci, similar immune-dysregulation profiles, and comparable patterns of chronic-relapsing inflammation^10–12^. This may be related to their shared genetic susceptibility and immune regulatory abnormalities. The evidence for this is that the polymorphisms in the human leukocyte antigen gene region are associated with an increased risk of AIH and MS^13,14^. Although there have been some reports on the co - occurrence of AIH and MS^15^, there is currently no detailed study on the correlation between the pathogenesis of the two diseases. Therefore, it is of great significance to study the common diagnostic and therapeutic targets for the two diseases.

In recent years, microarray technology and bioinformatics analysis have been widely used to screen genetic variations at the genomic level. In this study, for the first time, we utilized the gene expression data related to AIH and MS in the Gene Expression Omnibus (GEO) database^16^ and identified the common molecular targets of the two diseases through integrative analysis. First, we used volcano plots and heatmaps to display the differentially expressed genes in AIH and MS, and explored the biological pathways enriched by these genes through Gene Ontology (GO) and Kyoto Encyclopedia of Genes and Genomes (KEGG) pathway analysis^17^. Subsequently, potential common molecular targets of AIH and MS were determined through intersection analysis. These targets may play a crucial role in the pathogenesis of the two diseases. Protein - Protein Interaction (PPI) analysis was carried out to understand the protein - protein interaction relationships among various genes^18^. To further screen and verify the diagnostic efficiency of these targets, we employed the Least Absolute Shrinkage and Selection Operator (LASSO) regression model for screening^19^, and evaluated the diagnostic value of key targets through the Area Under the Curve (AUC) and Receiver Operating Characteristic (ROC) curve. In addition, to explore the potential therapeutic targets for the two diseases, we constructed a regulatory network of Hub Genes - Transcription Factors (TFs) - microRNA (miRNA) for analysis^20^. To explore the role of the common key gene Zinc Finger Protein Y - linked (ZFY) in the diseases, we also conducted single - gene immune infiltration analysis to understand its immunomodulatory role in the disease - onset process. Finally, to further verify the role of these targets in AIH and MS, we carried out animal experiments on C57BL/6 mice, observed the expression changes of these targets in the disease state, and preliminarily explored their potential functions in the occurrence and development of the diseases. The implementation of this study not only helps to reveal the potential molecular links between AIH and MS but also may provide new targets and ideas for the treatment of the two diseases.

## Methods

### Data collection and preparation

The data used in this study were obtained from the GEO repository (https://www.ncbi.nlm.nih.gov/geo/). The data for AIH were from GSE159676^21^, and the samples were liver tissues from normal controls or AIH patients. The data for MS were from two datasets, GSE131279 and GSE131281^22^, and the samples were brain tissues from normal controls or MS patients.

### Differential gene expression analysis

After preparing the data for the two diseases, the “limma” R (version 4.4.2) package was used to screen for differentially expressed genes (DEGs) between AIH, MS, and their respective control groups. The application criteria were a P - value < 0.05 and |log2FC| > 1. Subsequently, heatmaps and volcano plots were used to visually display the differential analysis results for each group.

### Functional enrichment analysis

In this study, we used an online platform to perform GO and KEGG enrichment analyses on the DEGs. This platform was developed based on the “clusterProfiler” and “pathview” packages and is available in both Chinese (https://www.bioinformatics.com.cn) and English (http://www.bioinformatics.com.cn/srplot) versions. First, the differentially expressed genes were input into the Chinese - version platform. In the platform settings, we selected the target species as human. The platform then automatically performed GO and KEGG enrichment analyses based on the input gene list.

### Protein - protein interaction network analysis

The two sets of DEGs were imported into Venny 2.1.0 (https://bioinfogp.cnb.csic.es/tools/venny/index.html) to screen for intersection genes. The STRING database (https://cn.string - db.org/) was used to construct the PPI network of the intersection genes(threshold to 0.4). Since the metabolic roles of the intersection genes in vivo have not been fully elucidated, they may be involved in the functional regulation of multiple organs and tissues.

To further analyze the tissue distribution of the intersection genes, the BioGPS^23^ database was used to obtain the mRNA expression levels of the relevant targets in organs and tissues. BioGPS provides normal human tissue data based on the U133plus2 Affymetrix chip, and the mRNA expression levels are represented as z - scores calculated by the barcode function of the R package frma. The top 10 related tissues or organs were selected according to the gene expression levels, and the screening results were imported into Cytoscape^24^ 3.9.1 to construct a network diagram of the distribution of intersection genes and tissues/organs.

### Construction of hub genes - TFs - miRNA regulatory network

The miRWalk database (https://mirwalk.umm.uniheidelberg.de/) was used to predict the miRNAs of the target genes. The screening parameters were set as Score ≥ 0.95, the target sites were limited to the 3UTR region, and the miRDB database was selected for verification (parameter set to 1). Subsequently, the iRegulon plugin in Cytoscape software was used to predict TFs. The normalized enrichment score (NES) > 5 was used as the screening threshold to ensure the accuracy and reliability of the results. Finally, the prediction results were integrated, and a TF - miRNA - target gene regulatory network was constructed through Cytoscape software (3.9.1) to visually display the regulatory relationships and further analyze the potential regulatory mechanisms.

### Feature selection using established machine learning algorithms

To identify the genes co - existing between AIH and MS, a detailed analysis was performed using the machine - learning algorithm of LASSO regression. To ensure the reproducibility of our findings, the seed number of the disease groups was set to 10. The first step was to input the previously identified 26 common genes into the LASSO algorithm for each disease group. Using the R package “glmnet”, we constructed a regression model with 10 - fold cross - validation. The “family” parameter was set to “binomial”, and the optimal λ value was determined by “λ.min”. Then, the log - curve of the LASSO coefficients of the 26 features was visualized. Next, the partial likelihood deviance (binomial deviance) and log(λ) curves were plotted. The best values of 1 se (1 - SE criterion) of the lowest standard were calculated, which were 4 for AIH and 6 for MS, respectively.

### Development and evaluation of the nomogram

To comprehensively evaluate the predictive performance and clinical application value of the model, we combined the nomogram, machine - learning results, and ROC analysis. First, key genes for each of the two diseases were screened through machine - learning algorithms, and a nomogram was constructed with the help of the “rms” R package to demonstrate the predictive efficacy of these genes. Then, ROC analysis was performed using the “pROC” R package. The diagnostic accuracy of these genes was evaluated using ROC curves, and the AUC value was calculated to quantify their discriminatory ability. Through this approach, we aimed to identify disease diagnostic targets with potential clinical application value and provide a scientific basis for subsequent diagnosis and treatment. Finally, the calibration curve was used to evaluate the prediction accuracy of the nomogram model.

### Immune infiltration analysis

The “GSVA” R package was used to perform single - sample gene - set enrichment analysis (ssGSEA) on ZFY gene, a diagnostic marker gene common to the two diseases, to evaluate the infiltration scores of 28 immune cells.

### Experimental animals

SPF - grade male C57BL/6 mice weighing 18 - 20 g were used in the experiment. The mice were purchased from Beijing Vital River Laboratory Animal Co., Ltd. (License No.: SCXK (Jing) 2016 - 0006) and were housed in a controlled environment with a 12 - h light - dark cycle, a temperature of 25 ± 1 °C, free access to water, and a normal diet. All animal experiments were approved by the Animal Use and Management Committee of Shanxi University of Chinese Medicine. (1) Establishment of the Concanavalin A (ConA) Animal Model: Twenty mice were first acclimatized for 7 days and then randomly divided into a normal group and a ConA group, with 10 mice in each group. Mice in the normal group were injected with normal saline via the tail vein, while mice in the ConA group were injected with 15 mg/kg of ConA (Solarbio, C8110, China) via the tail vein. (2) Establishment of the cuprizone (CPZ) Animal Model: Twenty mice were randomly and equally divided into a Normal group and a CPZ group according to their body weights. The Normal group was fed a normal diet for 12 weeks, while the CPZ group was fed a diet containing 0.2% CPZ (Sigma, 370 - 81 - 0, USA) for 10 weeks to establish a chronic demyelination mouse model. At the end of the 10th week, CPZ feeding was stopped, and the mice were fed a normal diet for another 2 weeks. During the entire experimental process, routine cage maintenance was carried out at least 3 times a week, and the daily status of the mice was observed and recorded.

### Behavioral tests

The mice were acclimated to the surroundings of the behavioral experiments in advance to avoid affecting the experimental results. (1) Open - Field Test (OFT): The mice were placed in a quiet and clean open - field facing the wall of the box. The activity of the mice in the open - field for 10 min was recorded using behavioral software. Four mice could be tested simultaneously each time. After the test, the open - field box was cleaned with 75% alcohol to exclude the interference of other factors, and then dried for the next group of experiments. (2) Elevated Plus - Maze (EPM) Test: The EPM consisted of two open arms and two closed arms of equal size intersecting each other. The mice were placed in the central area facing the open arm and with their backs to the experimenter. The behavior of the mice was recorded, and the maze was cleaned with 75% alcohol for the next group of experiments. (3) T - Maze Test: First, the mice were trained to be familiar with the left and right arms to identify the location of the bait arm. Then, the mice were fasted for 24 h before the experiment. They were placed in the main arm of the T - maze and allowed to move freely. The number of times and the distance the mice entered the bait arm were recorded to observe their working memory ability.

### Sample collection and specimen preparation

(1) Sample Collection from ConA Mouse Model: Eight hours after the tail - vein injection of ConA, the mice were anesthetized by intraperitoneal injection of pentobarbital sodium. After collecting blood, the mice were immediately sacrificed, and the serum was separated and stored at - 80 °C for subsequent experimental analysis. The liver and spleen were quickly dissected, gently rinsed with normal saline to remove residual blood and impurities on the surface, and then carefully blotted dry with filter paper. Subsequently, the weights of the liver and spleen were measured, and the tissues were divided into two parts: one part was fixed in 4% paraformaldehyde solution for histological analysis, and the other part was rapidly frozen and stored in a - 80 °C refrigerator for subsequent research. (2) Sample Collection from CPZ Mouse Model: After the behavioral experiments, 5 mice were randomly selected from each group and anesthetized with pentobarbital sodium (50 mg/kg). The chest was opened to expose the heart, and the right auricle was cut open. The heart was perfused with normal saline until the liver turned white, and then perfused with 4% paraformaldehyde. The brain tissue was dissected out, fixed in paraformaldehyde, and then dehydrated in 10%, 20%, and 30% sucrose solutions in gradient. After that, it was embedded with OTC, rapidly frozen in liquid nitrogen, and stored in a - 80 °C refrigerator for later use. When needed, the brain tissue was cut into 10 - μm - thick sections using a cryostat for pathological and immunofluorescence staining. Another 5 mice from each group were anesthetized in the same way as above, and only the heart was perfused with normal saline. The brain was removed, and the corpus callosum (CC) and the surrounding brain tissue of the mice were separated, weighed, placed in sterile EP tubes, and stored in a - 80 °C refrigerator for later use.

### Liver index

At the end of the experiment, the body weight and liver wet weight of the mice were recorded, and the liver index was calculated. The liver index was calculated according to the following formula: Liver Index (%) = Liver Mass (mg) / Body Weight (g) × 100. This index was used to evaluate the ratio of the liver to the body weight, providing a quantitative basis for experimental analysis.

### Biochemical detection and HE staining

The levels of alanine aminotransferase (ALT) and aspartate aminotransferase (AST) in the serum were determined using the ALT kit (mindrary, 105 - 020579 - 00, China) and the AST kit (mindrary, 105 - 020580 - 00, China), and detected using the BS - 240VET fully automated animal biochemical analyzer produced by Mindray (China). The right upper lobe of the liver tissue was taken, fixed in 4% paraformaldehyde solution, then dehydrated in an ethanol gradient, cleared with xylene, and embedded in paraffin to prepare tissue sections (4 - 5 μm thick). The sections were stained with hematoxylin - eosin (leagene biotech, DF0135, China), and after staining, they were mounted with neutral gum (Solarbio, g8590, China). The sections were observed under an optical microscope, and images were captured from randomly selected fields of view to analyze the pathological changes of the liver tissue.

### Luxol Fast Blue (LFB) and truegold staining detection

For Luxol Fast Blue staining(Solarbio,G3245, China), mouse brain sections were initially warmed and soaked in 70% ethanol for 15 minutes before being incubated in the staining solution at 56-60°C overnight. The next day, sections were thoroughly rinsed with 95% ethanol and deionized water to remove surplus stain, then treated with a mixture of 75% ethanol, deionized water, and 0.05% lithium carbonate to clearly differentiate gray and white matter. Following a series of ethanol dehydration steps and xylene clearing, the sections were mounted with neutral resin for microscopic examination.

For TrueGold staining (Ousaisi Biotechnology Co., Ltd,BK-AC001, China), brain slices were warmed and dried at 37°C for 30 minutes before diluting the stain and terminator in double-distilled water. The slices were then stained in the dark at 45°C for 30 minutes, rinsed with Phosphate Buffered Saline (PBS), and treated with the stain terminator at 45°C for 2-3 minutes. After three washes with PBS, the slices were dried, mounted, and imaged using a microscope.

### Immunofluorescence staining

The prepared frozen brain tissue sections were washed 3 times with PBS, 5 minutes each time. The primary antibody was added to a PBS solution containing 0.3% Triton X - 100 and 1% bovine serum albumin (BSA). After mixing, the solution was dropped onto the sections, and the sections were incubated overnight at 4 °C in a humid chamber. The next day, the sections were rinsed 3 times with PBS, 5 minutes each time. The corresponding species - specific secondary antibody was added and incubated at room temperature for 1 hour. Then, the sections were washed with PBS 3 times again. A fluorescence - quenching - resistant mounting medium containing DAPI was used for mounting. After 10 minutes, the results could be observed under a fluorescence microscope. Myelin basic protein (MBP, IF 1:200 dilution, Bio - Rad, aa82 - 87, China) in the CC region was detected. Finally, the fluorescence density was analyzed using Image J fiji 2.14.0.

### Reverse Transcription - Polymerase Chain Reaction (RT - PCR) detection

An appropriate amount of liver or brain tissue was placed in a mortar pre - cooled with liquid nitrogen. Liquid nitrogen was slowly added, and the tissue was continuously ground into powder with a pestle. The powder was then placed in an EP tube. Total RNA was extracted using the M5 Universal RNA Mini Kit (Mei5 Biotechnology Co., Ltd, MF036 - 01, China). After measuring the concentration, cDNA was synthesized using the PrimeScript™ RT reagent Kit (TaKaRa, RR047A, Japan). The TB Green Premix Ex Taq II Kit (TaKaRa, RR820A, Japan) was selected for real - time fluorescence quantitative PCR amplification. Finally, the relative expression levels of genes were calculated using the 2 - ΔΔCt formula to detect the gene expression levels of ZFY^25^, SLC22A15, EAF2^26^, ABCG2^27^, and HBB^28^ in the brain tissue. The primers used in this experiment were synthesized by Sangon Biotech (Shanghai, China) Co., Ltd. The primer sequences are shown in Table 1.

**Table 1:** Primers for RT-PCR.

Primers	Forwards sequence 5’-3’	Reverse sequence 5’-3’
ZFY	GACTAGACATGTCTTAACATCTGTCC	CCTATTGCATGGACAGCAGCTTATG
ABCG2	ATAGCCACAGGCCAAAGTGT	ACTGCAAAGCTGTGAAGCCA
HBB	TTTAACGATGGCCTGAATCACTT	CAGCACAATCACGATCATATTGC
EAF2	CCAGCGGGACTTGCATACC	GCCAACCTCAAGATTTCCTTCAC
SLC22A15	GGCACCGTCATCGGATCAG	CTCCACAGGCAGACCAGAAAA
GAPDH	TGTGTCCGTCGTGGATCTGA	TTGCTGTTGAAGTCGCAGGAG

### Statistical analysis

All statistical analyses were performed using R software (version 4.4.2) and GraphPad Prism 8.0.2. For all analyses, a *P* - value < 0.05 was set as statistically significant. Measurement data conforming to a normal distribution were expressed as x ± SD. For the animal experiment part, the Welch t - test was used for comparisons between two groups, and the Wilcoxon test was used for the rest.

## Results

### Identification of DEGs

**Fig. 1:**
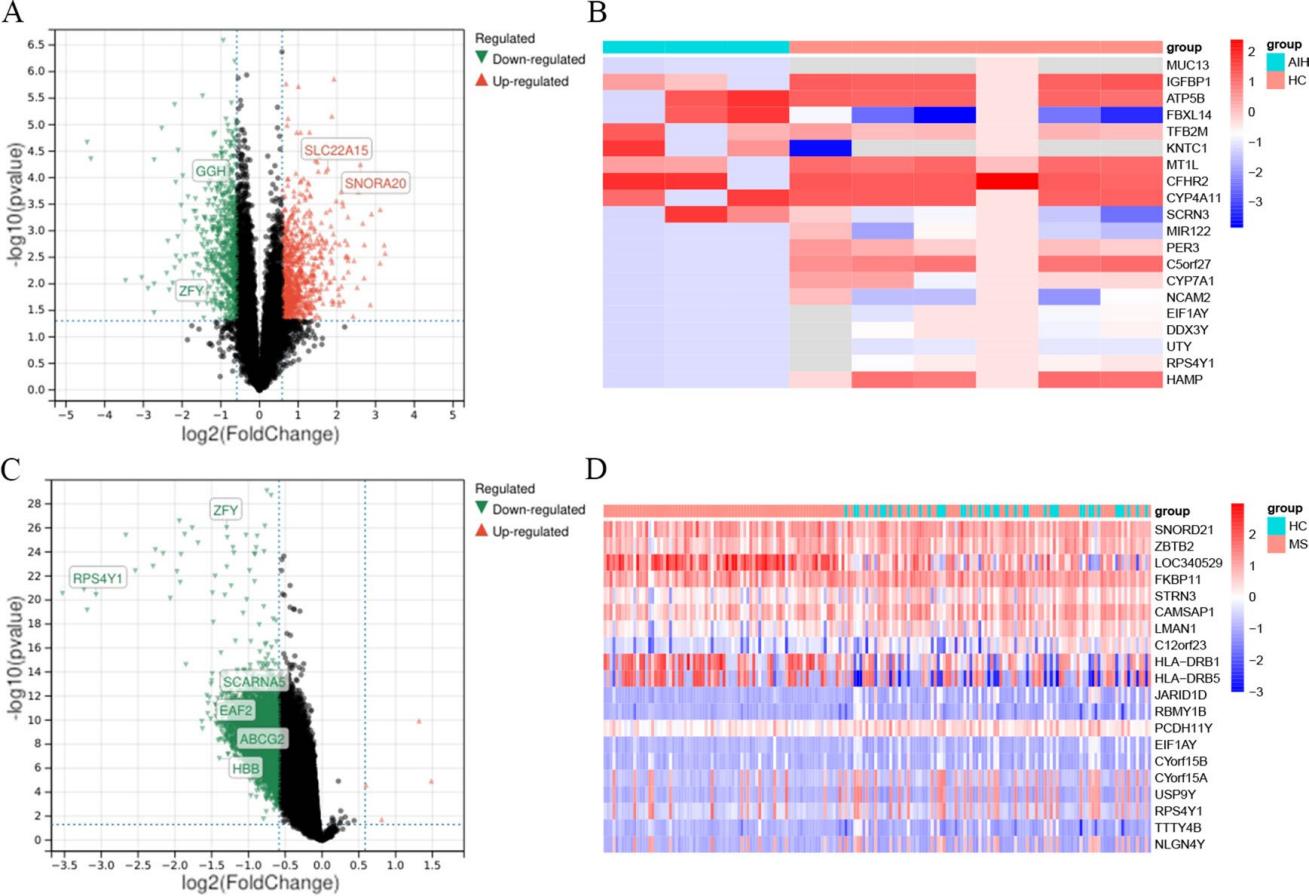
Screening results of DEGs (**A**). Volcano plot of differentially expressed genes in AIH. (**B**). Heatmap of differentially expressed genes in AIH. (**C**). Volcano plot of differentially expressed genes in MS. (**D**). Heatmap of differentially expressed genes in MS.

To investigate shared pathogenic mechanisms between autoimmune hepatitis (AIH) and multiple sclerosis (MS), we identified disease-specific transcriptional signatures. In AIH (GSE159676), 476 DEGs (296 upregulated, 180 downregulated) were detected, while in MS (integrated GSE131279/GSE131281), 857 DEGs were identified (3 upregulated, 854 downregulated). These were obtained from the combined datasets using the limma package for differential gene analysis in R (Fig. 1A, C). Similarly, they were obtained from the combined datasets using the pheatmap package (Fig. 1B, D).

### Enrichment analysis of DEGs

**Fig. 2:**
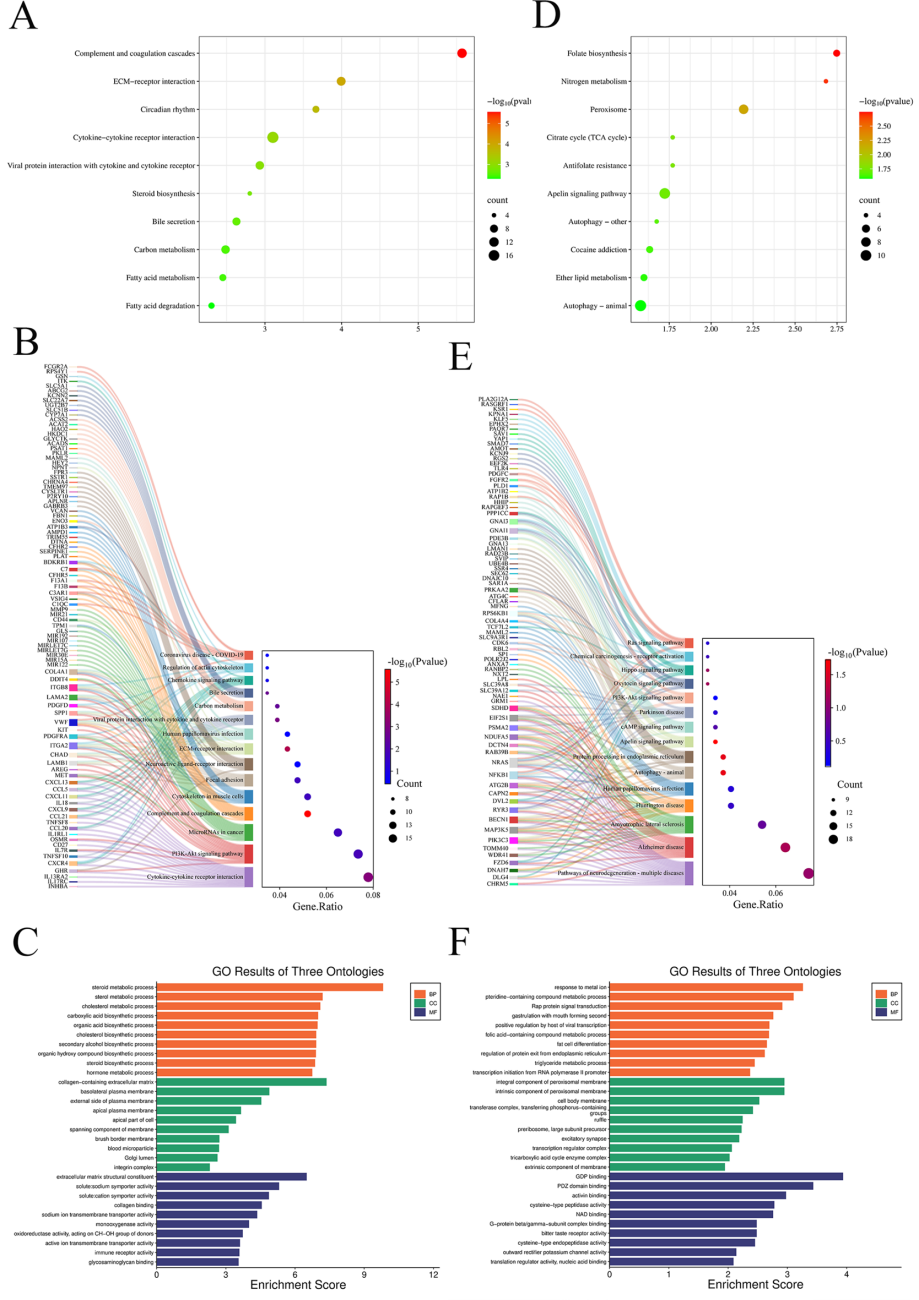
Results of GO enrichment analysis and KEGG enrichment analysis (**A**) and (**D**) show the KEGG enrichment analysis results of AIH and MS respectively, with bubble plots of the top 10 signaling pathways screened based on the P - value. (**B**) and (**E**) are the Sankey diagrams of the KEGG enrichment analysis results of AIH and MS respectively, with the top 15 signaling pathways screened according to the number of differentially - enriched genes. (**C**) and (**F**) are the GO enrichment analysis results of AIH and MS respectively, where the top 10 biological process (BP, orange bars), cellular component (CC, green bars), and molecular function (MF, light - blue bars) terms are shown separately.

The KEGG analysis^29^ results of AIH showed that in the bubble plot (Fig. 2A), the DEGs were mainly enriched in pathways such as Complement and coagulation cascades, ECM - receptor interaction, Cytokine - cytokine receptor interaction, and Circadian rhythm. In the Sankey diagram (Fig. 2B), according to the number of differentially - enriched genes, the DEGs were significantly enriched in pathways such as Cytokine - cytokine receptor interaction, Phosphatidylinositol 3 - Kinase/Protein Kinase B (PI3K/Akt) pathway, and MicroRNAs in cancer. These pathways are closely related to immune regulation and cell signaling. In the GO analysis (Fig. 2C), the top ten results of biological processes (BP), cellular components (CC), and molecular functions (MF) of AIH were presented respectively.

The KEGG analysis results of MS showed that in the bubble plot (Fig. 2D), the DEGs were mainly enriched in pathways such as Autophagy - animal, Apelin signaling pathway, and Peroxisome. In the Sankey diagram (Fig. 2E), according to the number of differentially - enriched genes, the DEGs were significantly enriched in neurodegenerative - disease - related pathways such as Pathways of neurodegeneration - multiple diseases, Alzheimer disease, and Amyotrophic lateral sclerosis, as well as the PI3K/Akt pathway and cAMP signaling pathway. This indicates that the gene changes in the MS model may be closely related to neurodegenerative diseases and autophagy regulation. In the GO analysis (Fig. 2F), the top ten results of BP, CC, and MF of MS were presented respectively.

### Construction of intersection gene PPI and organ - target network

**Fig. 3:**
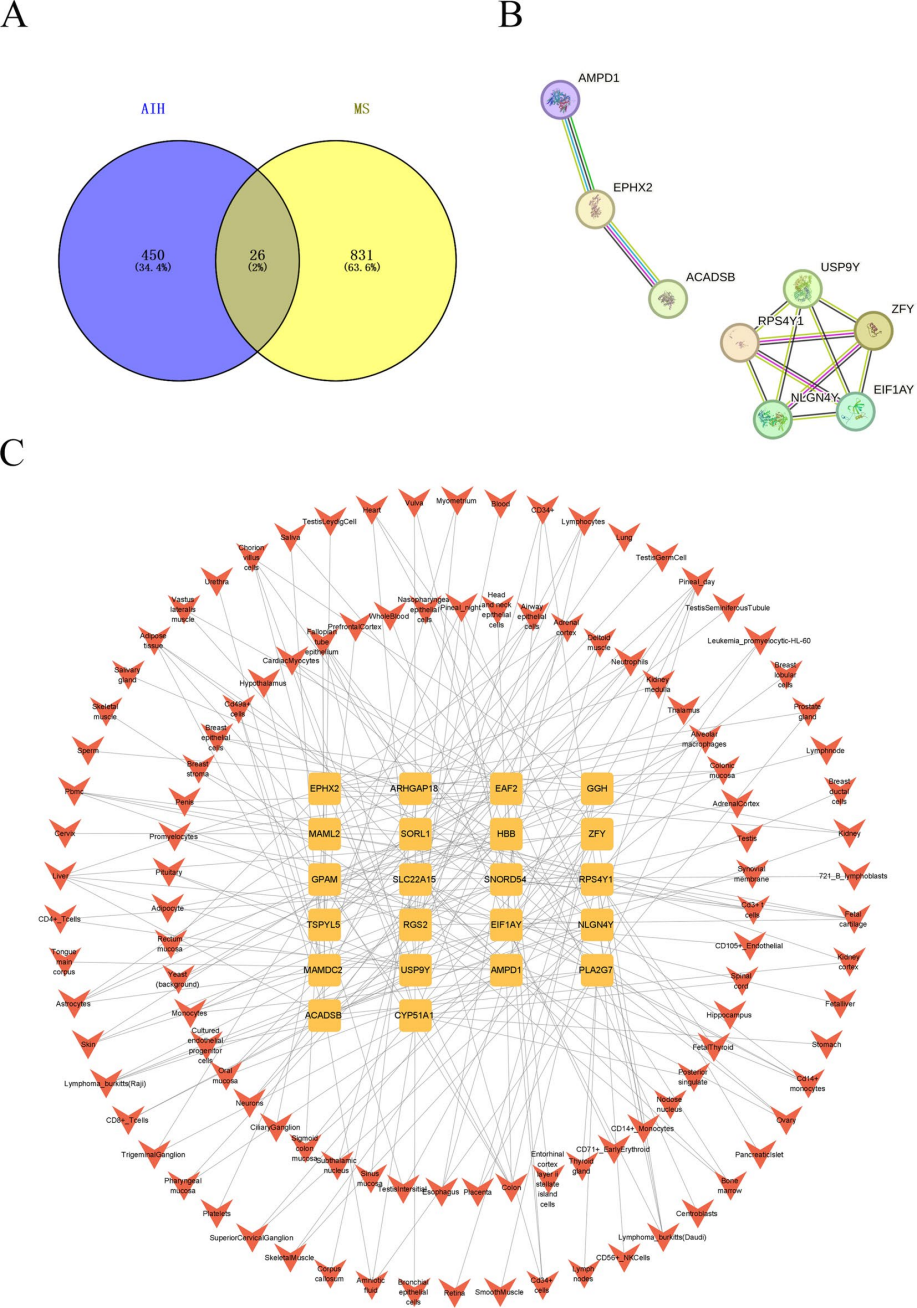
Construction results of intersection gene PPI and organ - target network (**A**). VENNY analysis diagram of intersection genes. It shows the intersection of differentially expressed genes in AIH and MS. (**B**). PPI network diagram of intersection genes. Nodes represent proteins, and edges represent interactions between proteins. (**C**). Organ - target association network diagram. Nodes represent targets, and edges represent associations between targets and organs.

Cross-disease comparison identified 26 shared DEGs (Fig. 3A). PPI network analysis revealed 8 tightly interconnected nodes (Fig. 3B), including ZFY and ABCG2—genes implicated in immune regulation and cellular metabolism. Organ-target mapping further prioritized the liver and colon as key sites of gene dysregulation (Fig. 3C), suggesting gut-liver-brain axis involvement in AIH-MS comorbidity.

### Discovery of common diagnostic biomarkers through machine - learning algorithms

**Fig. 4:**
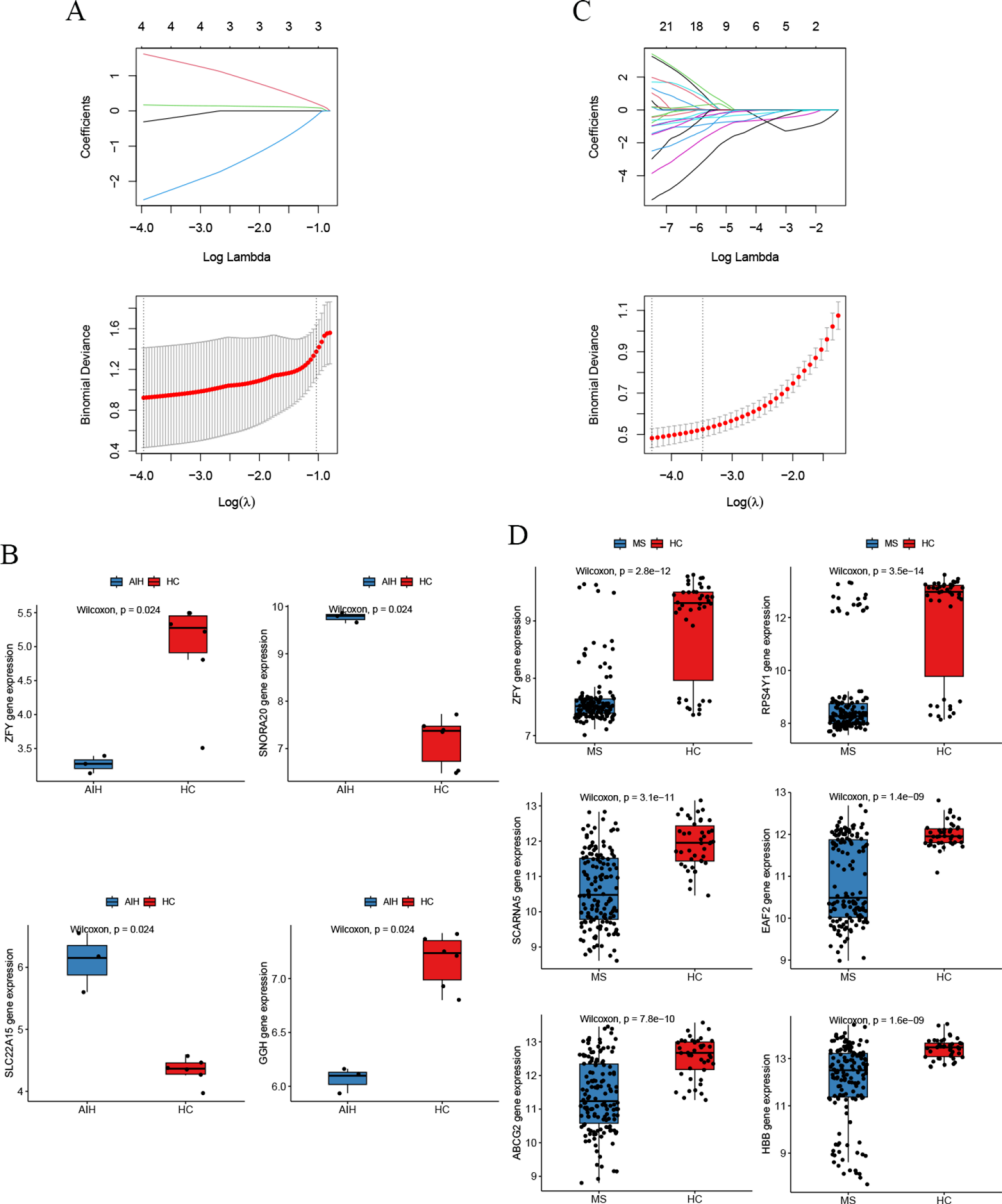
Results of screening diagnostic biomarkers by machine - learning algorithms (**A**). LASSO regression analysis result diagram of the AIH group. (**B**). Expression level diagram of potential diagnostic biomarker genes in the AIH group. (**C**). LASSO regression analysis result diagram of the MS group. (**D**). Expression level diagram of potential diagnostic biomarker genes in the MS group.

To further screen candidate diagnostic gene targets with significant eigenvalues for the classification of disease groups and control groups, based on the above - mentioned 26 common genes, the LASSO regression algorithm was used. In the AIH group, the LASSO regression algorithm identified 4 potential candidate genes that have a substantial impact on diagnosis (Fig. 4A), namely ZFY, SNORA20, SLC22A15, and GGH. Similarly, in the MS group, the LASSO algorithm identified 6 characteristic genes (Fig. 4C), namely ZFY, RPS4Y1, SCARNA5, EAF2, ABCG2, and HBB.

We examined the expression levels of potential diagnostic biomarker genes in AIH and MS. As shown in Fig. 4B, the expression of ZFY and GGH decreased in the AIH group, while the expression of SNORA20 and SLC22A15 increased. As shown in Fig. 4D, the expression levels of the 6 potential diagnostic biomarker genes decreased in the MS group. Notably, the expression of ZFY gene decreased in both the AIH group and the MS group.

### Construction and evaluation of nomograms and diagnostic models for key diagnostic biomarkers in AIH and MS

**Fig. 5:**
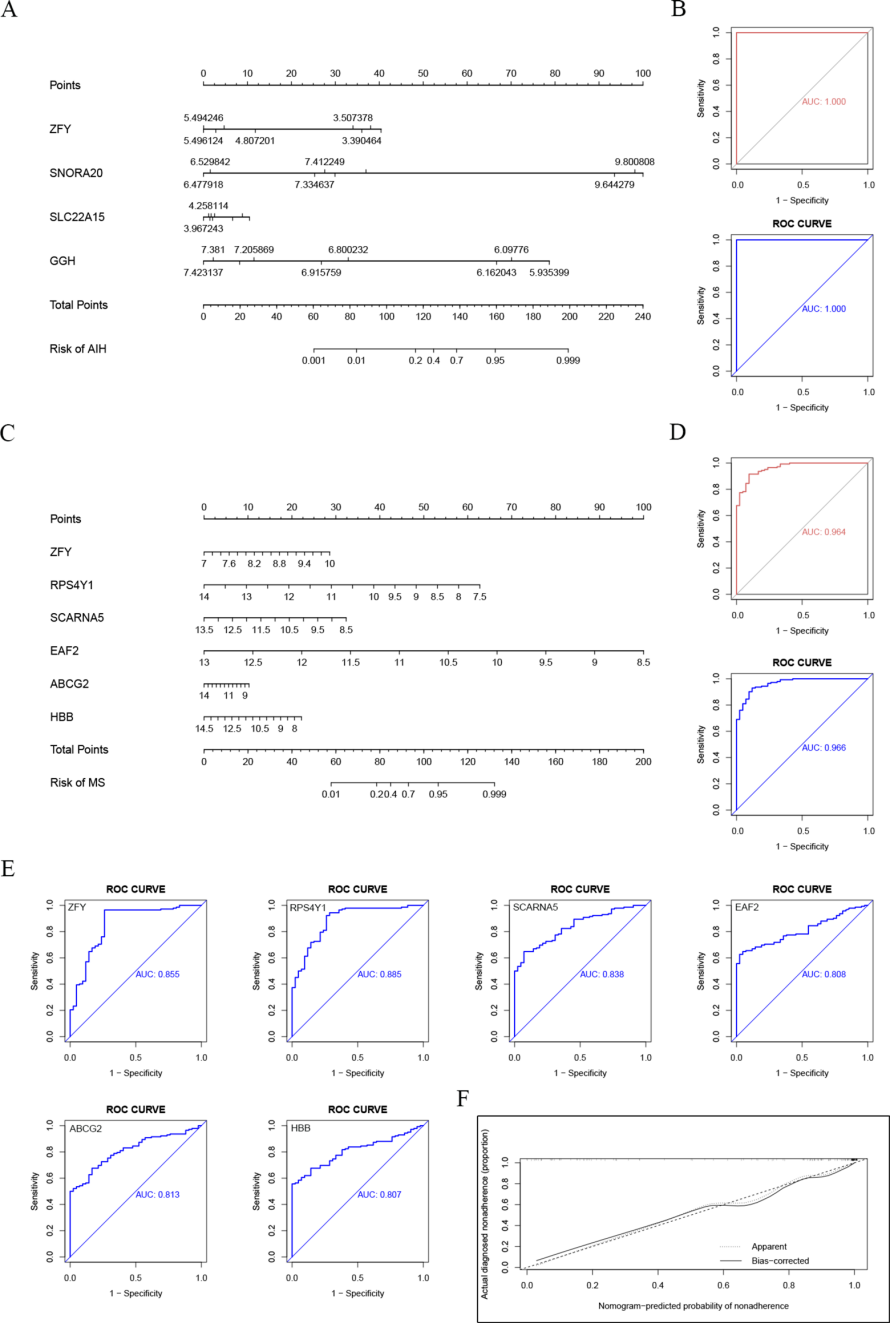
Construction and evaluation results of nomograms and diagnostic models (**A**). Nomogram of the AIH group (each gene corresponds to a score axis, the total score axis represents the comprehensive score of all genes, and the risk axis represents the disease risk predicted based on the total score). (**B**). ROC curve analysis diagram of the AIH group (the abscissa is the false - positive rate, and the ordinate is the true - positive rate. The closer the curve is to the upper - left corner, the higher the diagnostic efficiency. The AUC value represents the area under the curve, and the larger the AUC value, the higher the diagnostic efficiency). (**C**). Nomogram of the MS group. (**D**). ROC curve analysis diagram of the MS group. (**E**). Individual ROC analysis diagram of each gene in the MS group. (**F**). Calibration curve diagram of the MS group.

To comprehensively understand the diagnostic effects and significance of biomarker genes in AIH and MS, we constructed nomograms (Fig. 5A, C) and evaluated their diagnostic values using ROC curves. We performed machine - learning on the AIH group and the MS group respectively to draw AUC curves and further conducted ROC curve analysis (Fig. 5B, D) to evaluate the specificity and sensitivity of these gene sets in the diagnosis of AIH and MS. The test results were all far greater than the standard of 0.7, demonstrating their high predictive and discriminative abilities.

Next, we conducted individual ROC analysis of each gene and evaluated the performance of the nomogram. In the AIH group, the AUC values of key diagnostic biomarkers were all 1 (possibly due to the small sample size in the AIH group). In the MS group, ZFY (AUC = 0.855), RPS4Y1 (AUC = 0.885), SCARNA5 (AUC = 0.838), EAF2 (AUC = 0.808), ABCG2 (AUC = 0.813), and HBB (AUC = 0.807) all showed reliable predictive abilities (Fig. 5E). Figure 5F shows that the calibration curves in the training and validation groups closely coincide with the standard curve, indicating the high accuracy of the nomogram in predicting MS.

### Hub gene - TFs - miRNA regulatory network

**Fig. 6:**
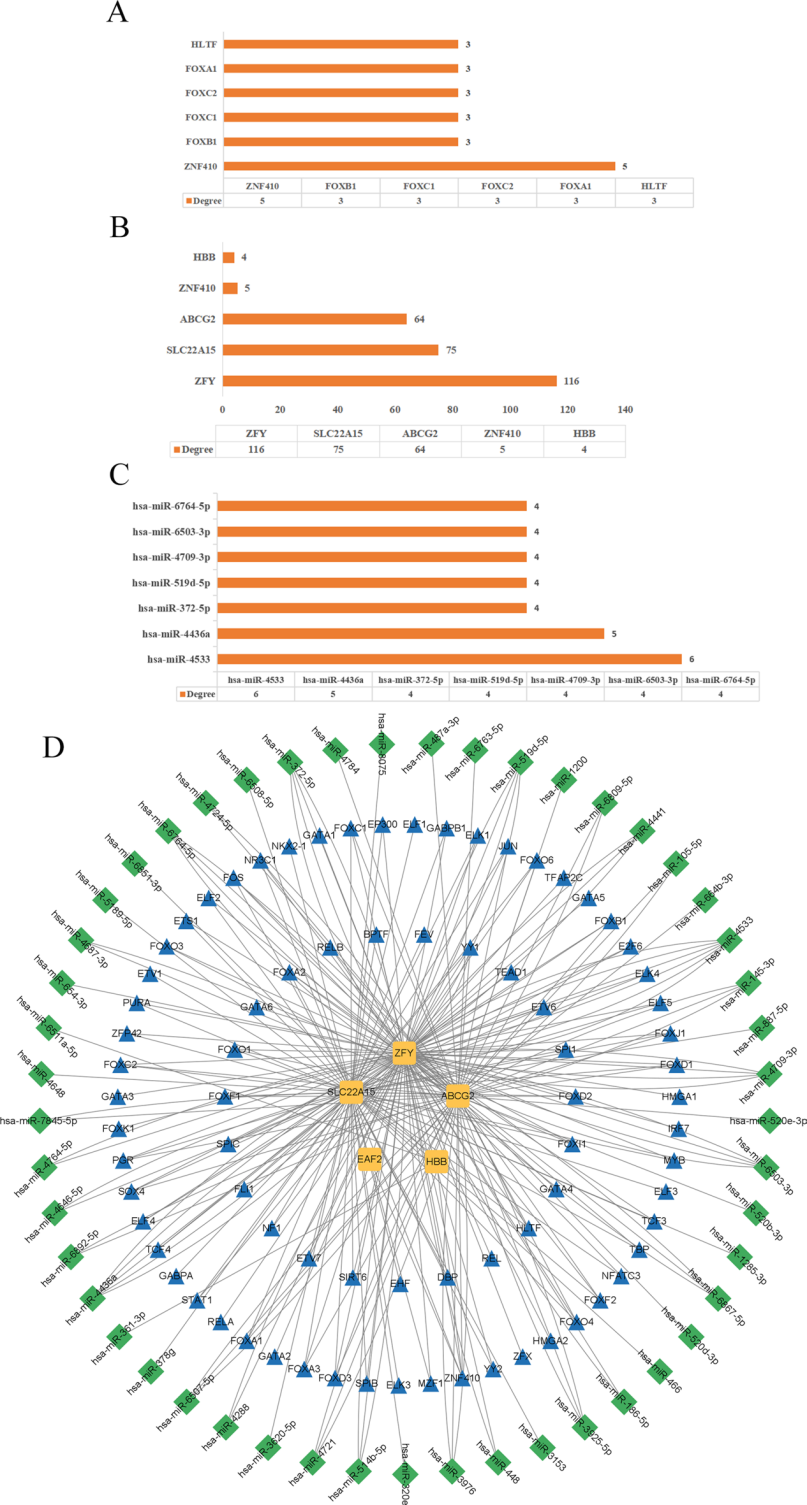
Construction results of the Hub Gene - TFs - miRNA regulatory network (**A**). The top 5 TFs in the regulatory network in terms of degree value. (**B**). The top 5 hub genes in the regulatory network in terms of degree value. (**C**). The top 7 miRNAs in the regulatory network in terms of degree value. (**D**). The candidate gene - TFs - miRNAs regulatory network diagram. Nodes represent candidate genes, TFs, and miRNAs, and edges represent the regulatory relationships between them.

The upstream regulatory mechanisms of candidate genes were preliminarily explored by predicting related TFs and miRNAs. Using the Cytoscape plugin iRegulon, 78 TFs were predicted. After screening and verification through the miRWalk 3.0 database, a total of 100 miRNAs were obtained. Network analysis showed that the top three TFs in terms of degree value were YY1, FOXB1, and FOXC1, indicating that they may have a core regulatory role in the regulatory network, as shown in Fig. 6A. In this network, the three genes with the highest degree values were ZFY, SLC22A15, and ABCG2, as shown in Fig. 6B. The top three miRNAs in terms of degree value were hsa - miR - 4533, hsa - miR - 4436a, and hsa - miR - 372 - 5p, suggesting that these miRNAs may play important functions in the regulation of candidate genes, as shown in Fig. 6C. After integrating the above - mentioned prediction results, a candidate gene - TFs - miRNAs regulatory network (Hub MitoDEGs - TFs - miRNAs regulatory network) containing 132 nodes and 260 edges was constructed, as shown in Fig. 6D. The construction of this network and the screening of its key nodes provide an important basis for further studying the upstream regulatory mechanisms of candidate genes and also lay a foundation for revealing their potential biological functions.

### Single - gene immune infiltration of the key diagnostic biomarker gene ZFY in AIH and MS

**Fig. 7:**
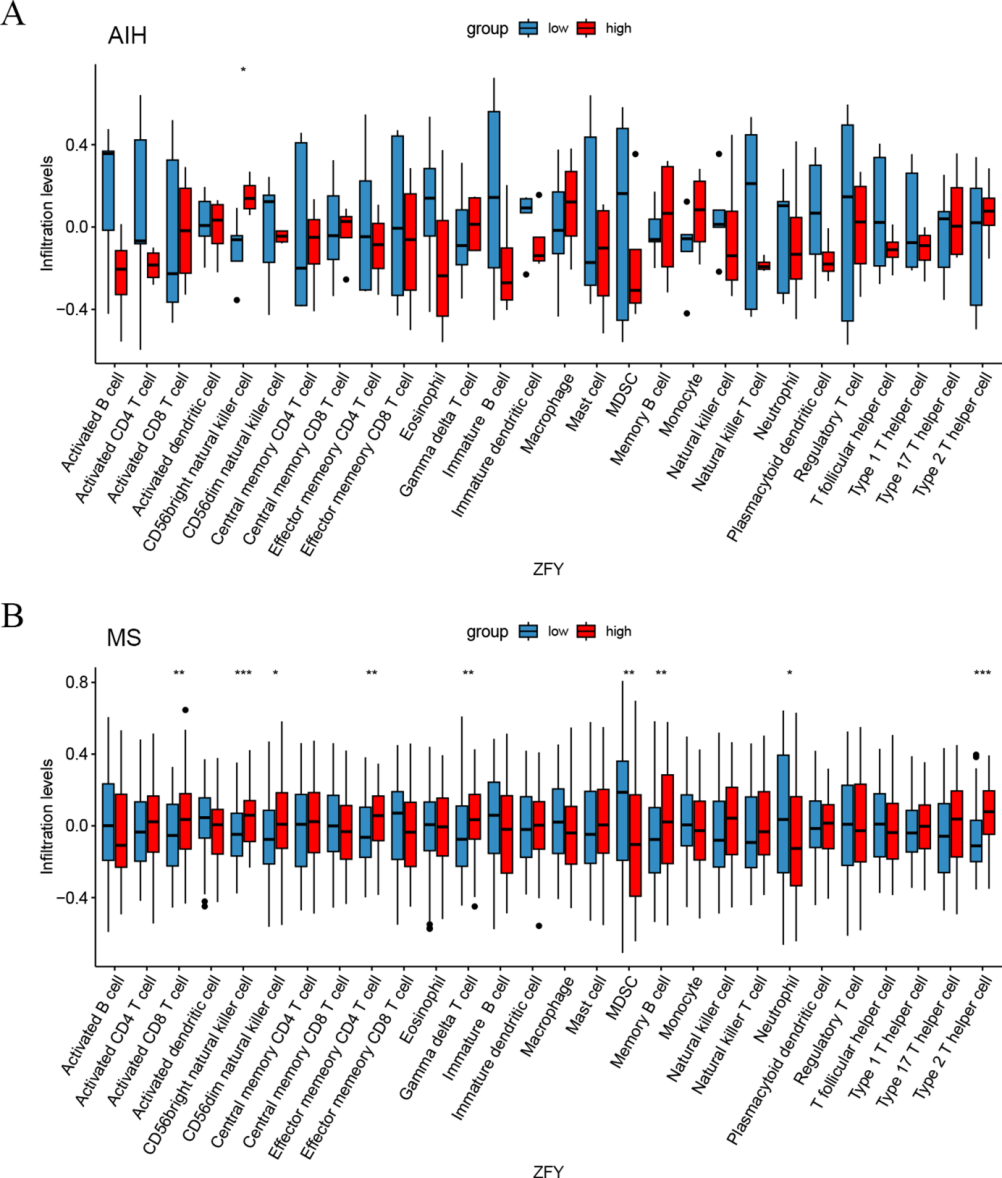
Results of single - gene immune infiltration analysis of ZFY gene (**A**). Comparison of immune cell infiltration levels between ZFY low - expression group and the high - expression group in AIH (The abscissa represents the type of immune cells, the ordinate represents the immune cell infiltration level. Columns of different colors represent different expression groups, and error bars represent the standard deviation. *P < 0.05, **P < 0.01, ***P < 0.001). (**B**). Comparison of immune cell infiltration levels between ZFY low - expression group and the high - expression group in MS.

In this study, the results of single - gene immune infiltration of ZFY in both AIH and MS showed that the immune infiltration level of CD56bright natural killer cell was significantly decreased in ZFY low - expression group (*P <* 0.05 and *P <* 0.001 respectively), as shown in Fig. 7A, B.

In addition, in the results of single - gene immune infiltration of ZFY in MS, the levels of Activated CD8 T cell (*P <* 0.01), CD56dim natural killer cell (*P <* 0.05), Effector memory CD4 T cell (*P <* 0.01), Gamma delta T cell (*P <* 0.01), Memory B cell (*P <* 0.01), and Type 2 T helper cell (*P <* 0.001) decreased in ZFY low - expression group, while the levels of Myeloid-derived suppressor cells (MDSC) (*P <* 0.01) and Neutrophil (*P <* 0.05) increased, as shown in Fig. 7B.

### Liver injury in ConA - treated mice

**Fig. 8:**
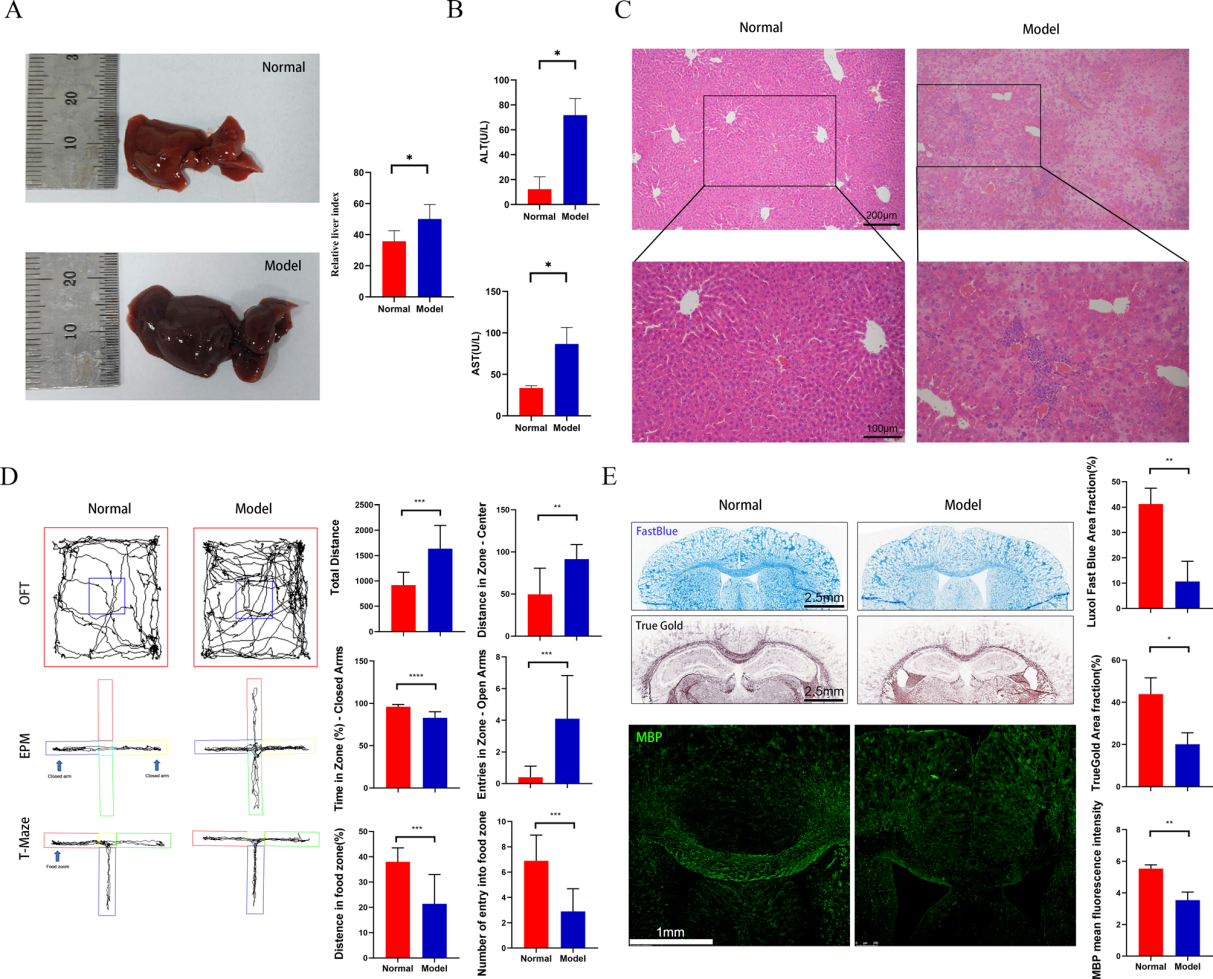
Results of animal experiments The appearance and liver index of ConA mice liver ($\overline{{\mathrm{X}}}$± SD, n=3) .*P <0.05, comparison between Control group and ConA group (Welch t-test). **B**. The levels of ALT and AST in the serum of ConA mice ($\overline{{\mathrm{X}}}$ ± SD, n=3) . *P <0.05, comparison between Control group and ConA group (Welch t-test). **C**. HE staining results of ConA mouse liver tissue (Magnification 10×/20×, Scale Bar=200 μm/100 μm) .**D**. The behavioral performance (OFT, EPM and T-Maze) of CPZ mice($\overline{{\mathrm{X}}}$±SD, n=10). ** *P <*0.01, *** *P <*0.001, **** *P <*0.0001, comparison between Normal group and CPZ group(Welch t-test). E. The Histological Staining with LFB (Scale Bar = 2.5 mm, $\overline{{\mathrm{X}}}$ ±SD, n=3), TrueGold (Scale Bar = 2.5 mm, $\overline{{\mathrm{X}}}$ ±SD, n=3) and the Immunofluorescence Staining of MBP (green, Magnification 5×, Scale Bar = 1 mm, $\overline{{\mathrm{X}}}$±SD, n=3) in CPZ mice CC. * *P <*0.05, ** *P <*0.01, comparison between Normal group and CPZ group(Welch t-test).

Compared with controls, ConA-treated mice exhibited markedly enlarged, dark-red and brittle livers with a significantly elevated liver index (*P* < 0.05, Fig. 8A), alongside sharply increased serum ALT and AST levels (both *P* < 0.05, Fig. 8B), indicating severe hepatic injury. The results of HE staining showed obvious inflammatory cell infiltration in the liver tissue of the ConA group, especially in the portal vein and interlobular septum regions. At the same time, a large number of liver cell structures were damaged, showing signs of severe necrosis and apoptosis (Fig. 8C). The above results fully demonstrate the successful establishment of the model group.

### Behavioral changes and demyelination in CPZ mice

Given the possibility of cognitive and behavioral disorders, including memory impairment and anxiety, in the CPZ mouse model, we evaluated the behavior of CPZ mice (Fig. 8D). In the OFT, compared with the Normal group, the total movement and central area activity of the CPZ group increased (*P <* 0.001, *P <* 0.01), indicating hyperactivity and anxiety. The EPM was used to evaluate anxiety - related behaviors, and the results showed that compared with the Normal group, the CPZ group spent less time in the closed arms and had more entries into the open arms (*P <* 0.0001, *P <* 0.001). The T - Maze test was used to evaluate spatial learning and memory, and the results showed that compared with the Normal group, the number of entries and the activity distance of CPZ - treated mice into the bait arm were significantly reduced (both *P <* 0.001). Furthermore, we performed pathological and immunofluorescence staining of myelin. LFB and TrueGold staining showed (Fig. 8E) that compared with the Normal group, the myelin staining in the CC region of the CPZ group was significantly decreased (*P <* 0.01, *P <* 0.05). Similarly, compared with the Normal group, the expression of MBP in the CPZ group was significantly decreased (*P <* 0.01). The above results all prove the successful establishment of the CPZ mouse model.

### RT - PCR detection

**Fig. 9:**
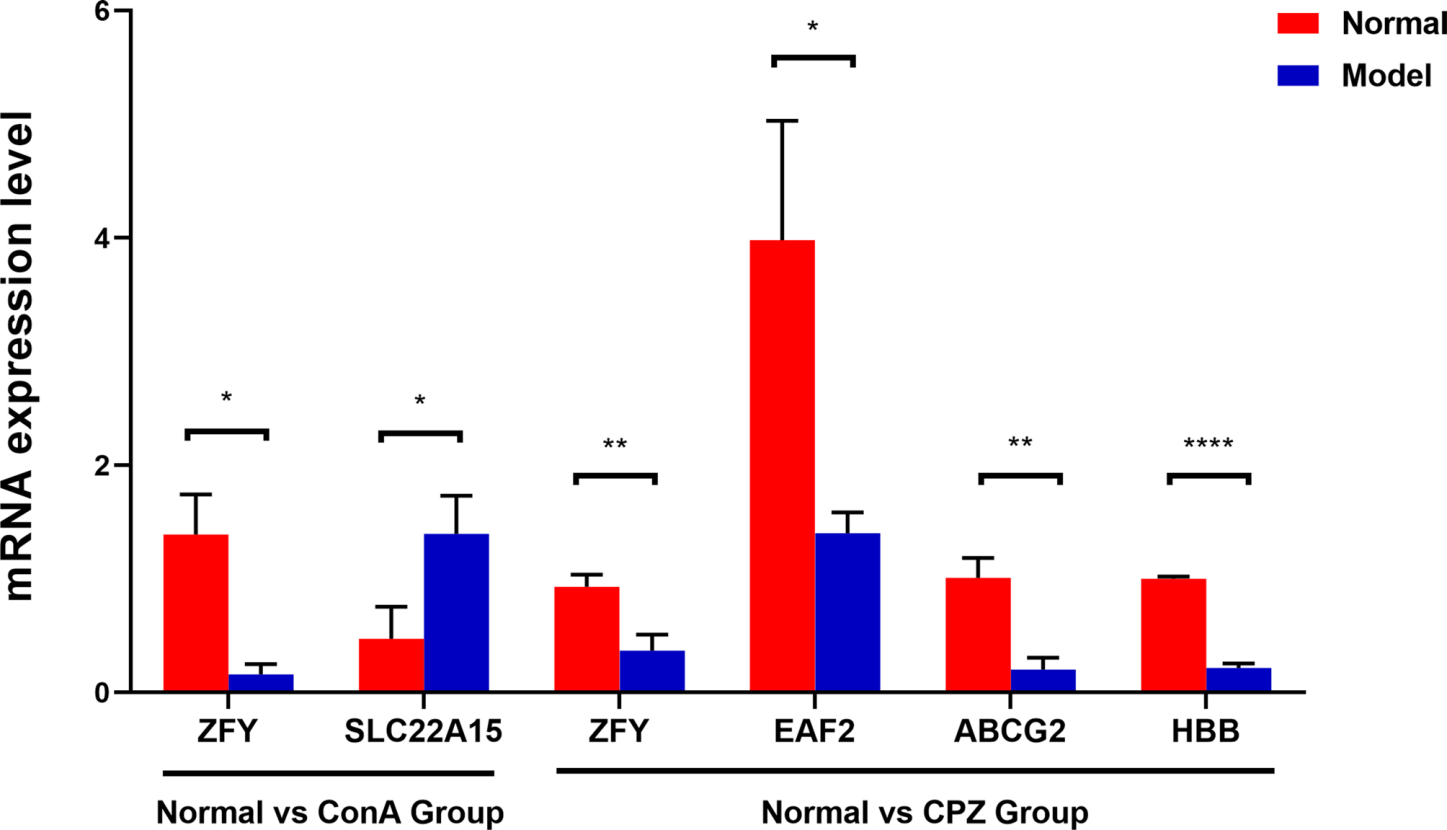
RT-PCR detection results of gene expression levels RT-PCR detection results of ConA mice liver and CPZ mice brain($\overline{{\mathrm{X}}}$±SD, n=10). * *P <*0.05, ** *P <*0.01, **** *P <*0.0001, comparison between Normal group and Model(ConA/CPZ) group(Welch t-test).

To verify the differences in gene expression levels among ConA - treated mice, CPZ - treated mice, and Normal mice, we performed RT - PCR detection. The results showed (Fig. 9) that compared with the Normal group, the expression level of ZFY gene in ConA - treated mice was significantly decreased, and the expression level of the SLC22A15 gene was significantly increased (both *P <* 0.05). Similarly, compared with the Normal group, the expression levels of ZFY, EAF2, ABCG2, and HBB genes in CPZ - treated mice were significantly decreased (*P <* 0.01, *P <* 0.05, *P <* 0.01, and *P <* 0.0001 respectively), which was consistent with the results of our bioinformatics analysis.

## Discussion

In this study, we conducted a series of bioinformatics analyses on the gene chip databases of AIH and MS, and obtained the respective DEGs of AIH and MS based on the GEO database. The GO enrichment analysis of the DEGs of the two diseases showed that the PI3K/Akt pathway was significantly enriched in the KEGG analysis of both AIH and MS, suggesting that it may be a common key regulatory mechanism for the two diseases. In addition, Cytokine - cytokine receptor interaction and ECM - receptor interaction were significantly enriched in AIH and also showed certain correlations in MS, further indicating the important roles of these pathways in inflammation regulation and cellular microenvironment control. These results provide important clues for further studying the common pathological mechanisms of AIH and MS.

Subsequently, we took the intersection of the DEGs of AIH and MS and obtained 26 intersection genes. By constructing an organ - target network of these intersection genes, it was highly suggestive that the mRNA levels of 22 targets were significantly increased in specific organs. These included fallopian tube epithelium, liver, pineal gland (daytime), colon, and Burkitt lymphoma cells. For AIH, apart from the liver itself, the colon and Burkitt lymphoma cells among the targets are likely to be directly involved in the initiation and continuous amplification of the local immune - inflammatory response in the liver. These targets may, through regulating multiple processes such as the metabolism of liver cells, the recruitment and activation of immune cells in the liver, and the inter - cellular communication in the liver microenvironment, jointly promote the damage and pathological progression of liver tissue^30^. Similarly, in MS, changes in targets in related organs such as the pineal gland (daytime) and colon may be related to immune abnormalities in CNS, glial cell dysfunction, and neurotransmitter metabolic imbalance, thus affecting the normal physiological functions of CNS and triggering typical pathological changes such as demyelination. Studies have shown that the gut microbiota helps the pineal gland in the brain produce melatonin^31^, and melatonin shows preventive and therapeutic effects on various diseases, including neurological diseases such as Alzheimer’s disease, Parkinson’s disease, and MS^32^.

Then, we used machine - learning algorithms to identify diagnostic biomarkers with significant discriminatory power. The LASSO regression algorithm was used to evaluate 9 biomarker genes. Four common biomarkers were identified in the AIH group, and six biomarkers were identified in the MS group, among which ZFY was their common diagnostic biomarker gene. ROC curve analysis confirmed the diagnostic potential of these genes in distinguishing between the disease group and the control group. The AUC values indicated their good diagnostic efficacy, providing new candidate gene biomarkers for early clinical diagnosis. In combination with clinical practice, the detection of thses gene biomarkers may assist doctors in finding abnormalities in the early, asymptomatic stage of the disease, which is helpful for formulating personalized monitoring and intervention plans, improving patient prognosis, and reducing the disability rate of the disease.

We used these 9 biomarker genes to construct a Hub gene - TFs - miRNAs regulatory network, revealing the relationships among TFs, miRNAs, and Hub genes. TFs and miRNAs may play important roles in the pathogenesis of AIH and MS. They affect the functions and activities of immune cells by regulating gene expression and signaling pathways, thus participating in the disease process. Among the TFs, YY1, FOXB1, and FOXC1 ranked in the top three in terms of degree value. As TFs, they may be involved in the pathogenesis of AIH and MS by regulating the expression of genes related to these diseases. These genes may be involved in immune responses, inflammatory processes, cell proliferation, and apoptosis. They may also affect disease progression by regulating specific signaling pathways, such as PI3K/Akt. In terms of miRNAs, hsa - miR - 4533, hsa - miR - 4436a, and hsa - miR - 372 - 5p had the highest degree values. These miRNAs bind to the mRNAs of candidate genes through base - pairing complementarity, either inhibiting the translation process or promoting mRNA degradation, thereby achieving negative regulation of gene expression. However, the specific mechanisms of action of these molecules in diseases still need further in - depth study.

Based on the above results, we found that ZFY is a common diagnostic biomarker gene for both AIH and MS. We noticed that ZFY gene was low - expressed in the disease groups of both diseases and high - expressed in the normal group. Immune infiltration analysis revealed a close connection between ZFY gene and immune cell infiltration. In both AIH and MS, the infiltration level of CD56bright natural killer cells was significantly decreased in ZFY low - expression group, indicating its crucial role in maintaining immune surveillance and immune defense functions^33^. The lack of its expression may weaken the body’s ability to clear diseased cells^34^. In MS, the imbalance of infiltration of multiple immune cells such as Activated CD8 T cells and CD56dim natural killer cells reflects the complex disorder of the immune microenvironment in CNS. The increase in MDSC and the decrease in Memory B cells, Effector memory CD4 T cells, etc. jointly indicate abnormal immune cell functions and disruption of immune tolerance, which are likely to promote the progression of neuroinflammation and myelin damage. This further emphasizes the central position of ZFY gene in the immune regulation network, providing key cellular targets and clues to the mechanism of action for immunotherapy intervention. Clinically, immunomodulatory therapies can be explored based on this, such as immunocytokine therapy or the application of immune checkpoint inhibitors, to restore immune homeostasis and reduce tissue damage by correcting immune cell infiltration and functional abnormalities. This finding not only reveals the importance of ZFY gene in maintaining normal immune function, but also provides a new perspective for us to understand the pathogenesis of AIH and MS. ZFY gene is located on the Y chromosome and plays a key role in sex determination, germ cell development, and gene expression regulation in organisms^35^. Therefore, ZFY gene is expected to become a gene target for diagnosing male patients with AIH or MS.

In addition, there is a close relationship between the PI3K/Akt pathway obtained from the KEGG analysis and CD56bright NK cells. The PI3K/Akt pathway is a crucial intracellular signal transduction pathway that is widely involved in multiple physiological processes such as cell proliferation, survival, metabolism, and angiogenesis, especially in the specific immune cell subset of CD56bright NK cells^36,37^.

Specifically, the activation of the PI3K/Akt pathway promotes the proliferation and survival of CD56bright NK cells. This effect is achieved through Akt, a downstream effector molecule of PI3K. When Akt is phosphorylated and activated, it can further phosphorylate various downstream substrates. Among these substrates are proteins closely related to cell survival and proliferation. Studies have shown that IL - 15 can activate the PI3K/Akt pathway in CD56bright NK cells, and this pathway is crucial for the anti - tumor response of CD56bright NK cells induced by IL - 15. PI3K inhibitors can significantly impair the effect of IL - 15 on CD56bright NK cells, indicating the key role of the PI3K/Akt pathway^38^. This regulatory mechanism is essential for maintaining the number and function of CD56bright NK cells.

The PI3K/Akt pathway is also involved in the functional regulation of CD56bright NK cells. By activating this pathway, the recognition and killing ability of NK cells against target cells can be enhanced, and the production of cytokines such as IFN - γ can be promoted. Some studies have also shown that the PI3K/Akt pathway may indirectly participate in the regulation of the functions of CD56bright NK cells. Although it does not directly elaborate on its effects on proliferation and survival, its importance in related physiological processes can be seen from the overall research^39^. The enhancement of these effector functions enables CD56bright NK cells to play a more active role in the body’s immune response.

To further verify the diagnostic effects of ZFY, SLC22A15, EAF2, ABCG2, and HBB, we conducted animal experiments. In the AIH mouse model, ConA induced liver enlargement, elevated transaminases, and pathological changes in liver tissue. In the CPZ - induced MS mouse model, the results of behavioral tests and myelin staining fully demonstrated that the model mice exhibited typical cognitive impairment, anxiety, and demyelination. Finally, we used RT - PCR to verify the changes in the gene expression levels of the above - mentioned five genes in their respective diseases, once again verifying their diagnostic efficiency.

However, this study has certain limitations. Bioinformatics analysis relies on data from public databases, and sample heterogeneity and data bias are inevitable; additionally, the MS transcriptomes were female-predominant, whereas the AIH series and our animal models were male-only, so the clinical relevance of ZFY down-regulation is at present restricted to male patients. Animal experiments have only preliminarily revealed the expression changes of ZFY gene in disease models, and the detailed molecular mechanism of its action still needs further analysis and verification, such as using gene-edited animal models (ZFY gene knockout or overexpression mice), as well as further clinical experiments to elucidate its causal relationship in disease occurrence and development.

## Conclusion

Our comprehensive bioinformatics analysis and experimental validation suggest that ZFY may be a potential biomarker for AIH and MS, and its downregulation is associated with disease status. Our data highlights the relationship between ZFY, PI3K/Akt pathways, and immune infiltration processes. However, these findings should currently be viewed as hypothesis generation rather than conclusive. Future research will use gene edited animal models and larger clinical cohorts, which are crucial for validating the diagnostic utility and mechanistic role of ZFY in these complex autoimmune diseases.

## Data Availability

The gene expression data utilized in this study, including those for Autoimmune Hepatitis (AIH) from the GSE159676 dataset and for Multiple Sclerosis (MS) from the GSE131279 and GSE131281 datasets, were sourced from the publicly accessible Gene Expression Omnibus (GEO) database (https://www.ncbi.nlm.nih.gov/geo/). These datasets are openly available for researchers to access and analyze. All constructs created in this work are available from the corresponding authors.

## Declarations

### Animal guidelines

All applicable institutional and national guidelines for the care and use of animals were followed. The study was conducted in accordance with the ARRIVE guidelines. All animal experimental procedures were approved by the Animal Use and Management Committee of Shanxi University of Chinese Medicine (Docket # AWE202403147; 2019LL106).
